# Supplementary material for: Using the theory of change (ToC) to co-design a primary mental healthcare system for older people with common mental disorders in Hong Kong
Source: Glob Ment Health (Camb). 2026 Mar 11;13:e59. doi: 10.1017/gmh.2026.10163 (PMC13112318; doi:10.1017/gmh.2026.10163)
Supplement: Liu et al. supplementary material [file S2054425126101630sup001.docx]

Supplementary Table 1. Details of the Rapid Situation Analysis (RSA) results shared with Theory of Change (ToC) workshop participants

| Areas of interest for discussion | Key findings |
| --- | --- |
| (1) Structure of Hong Kong’s population | - An increase in the population of older people for the next 50 years - 18.4% ≥ 65 in 2019 → aged society - 38.4% ≥65 in 2069 → super-aged society - An increase in life expectancy for both males and females - Male: 82.2 in 2019 → 88.4 in 2069 - Female: 88.1 in 2019 → 93.9 in 2069 - An increase in median age - 45.5 in 2019 → 57.4 in 2069 - An increase in elderly dependency ration - 26.5 in 2019 → 71.2 in 2069   (Census and Statistics Department of Hong Kong Special Administrative Region, 2020) |
| (2) The prevalence of CMD in older people | - Globally - Major depressive disorder (MDD): 13.3% (range: 1.1% to 23.3%) - Anxiety disorders: 0.8% to 5.4% (subthreshold: 1.1% to 7.9%) - Hong Kong - Around 1 in 9 older people experience some CMD   (Abdoli et al., 2022; Witlox et al., 2021; Lam et al., 2015) |
| (3) The impacts of CMD on individuals and society | - Higher depressive symptom severity is associated with higher care utilisation, care expenditures, predicted care expenditure, and incremental cost (Lu et al., 2021) - Preventing depression in later life is a crucial part of suicide prevention (De Leo, 2022) |
| (4) Mental healthcare workforce | - Hong Kong (Service providers: total number (number per 100,000 population) - Medical practitioners: 15,013 (202.8) - Psychiatrists: 428 (5.8) - Psychiatric nurses: 3,475 (46.9) - Clinical psychologists: 622 (8.4) - Social workers: 26,660 (360.1) - Occupational therapists: 2,778 (37.5) - Physiotherapists: 3,949 (53.3) - Comparison of service providers across countries (Full-time equivalent per 100,000 population)  \|  \| Hong Kong (2022) \| Singapore (2021) \| Japan \| London (2021) \| \| --- \| --- \| --- \| --- \| --- \| \| Psychiatrist \| 5.8 \| 4.6 \| 23.5 (2014) \| 27.6 \| \| Psychiatric nurse \| 46.9 \| - \| 0.8 (2022) \| 43.6* \| \| Clinical psychologist \| 8.4 \| 9.7 \| 25.6 (2018) \| 83.9# \|   * Nurses with a mental health area of work or speciality/care setting  #Staff in applied psychology and psychological therapy |
| (5) Pathways to mental healthcare in the existing system | - 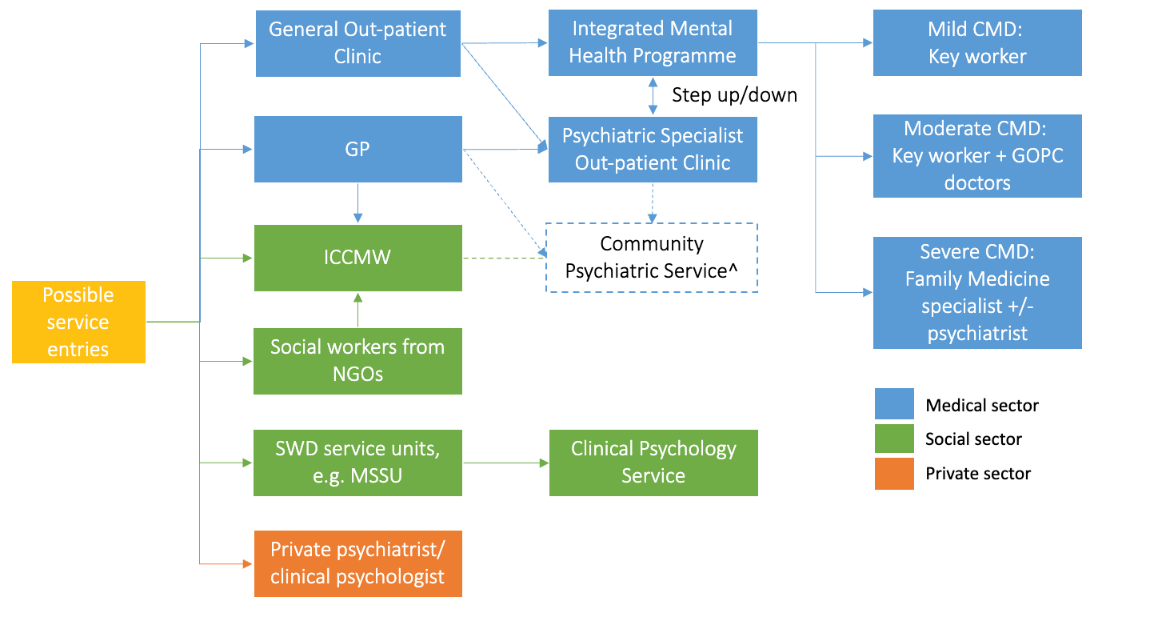 - Waiting time for new case booking at Psychiatry Specialist Out-patient clinics - In the past 12 months (1 January 2021 - 31 December 2021), there were 53,175 new case bookings. Waiting time ranges from < 1 week to 98 weeks depending on triage categories and service clusters  \|  \| New case bookings# \| \| Waiting time \| \| \| --- \| --- \| --- \| --- \| --- \| \|  \| Number \| Percentage \| \| Urgent case \| 3,144 \| 6% \| Median \| <1 week to 1 week \| \| Semi-urgent case \| 10,957 \| 21% \| Median \| 3 to 5 weeks \| \| Stable case \| 39,041 \| 73% \| Longest* \| 42 to 98 weeks \|   *The longest (90^th^ percentile) waiting time implies that appointments are earlier than the indicated time in 90% of the new bookings  #Exclusing cases pending for triage |
| (6) Policy regarding mental healthcare for older people | - Lift needy elderly out of poverty by providing case and non-cash welfare - Promote ageing in place by increasing service places for day care and home care services - Increase supply and service quality of subsidised and private residential care homes for the elderly - Promote the use of gerontechnology - Commence territory-wide mental health prevalence surveys covering elderly persons   (The Chief Executive’s 2021 policy address, 2021) |
| (7) Stakeholders involved from a bottom-up approach | - Decision makers - Policymakers (Health Bureau, Labour and Welfare Bureau, Hospital Authority, Department of Health, Social Welfare Department) - Philanthropy organisation managers - Service providers - General physician - Psychiatrist - Psychiatric nurse - Clinical psychologist - Social worker - Occupational therapist - Psychical therapist - Service recipients - Persons at risk of or with CMD - Carers of people with CMD - Relevant members of the public |

References:

Aboli, N., Salari, N., Darvishi, N., Jafarpour, S., Solaymani, M., Mohammadi, M., & Shohaimi, S. (2022). The global prevalence of major depressive disorder (MDD) among the elderly: A systematic review and meta-analysis. *Neuroscience & Biobehavioral Reviews. 132,* 1067-1073. https://doi.org/10.1016/j.neubiorev.2021.10.041

Census and Statistics Department of Hong Kong Special Administrative Region (HKSAR) (2020). *Hong Kong population projects 2020-2069*. <https://www.statistics.gov.hk/pub/B1120015082020XXXXB0100.pdf>

De Leo, D. (2022). Late-life suicide in an aging world. *Nature Aging, 2*, 7–12. <https://doi.org/10.1038/s43587-021-00160-1>

Lam, L. C., Wong, C. S., Wang, M. J., Chan, W. C., Chen, E. Y., Ng, R. M., Hung, S. F., Cheung, E. F., Sham, P. C., Chiu, H. F., Lam, M., Chang, W. C., Lee, E. H., Chiang, T. P., Lau, J. T., van Os, J., Lewis, G., Bebbington, P. (2015). Prevalence, psychosocial correlates and service utilization of depressive and anxiety disorders in Hong Kong: the Hong Kong Mental Morbidity Survey (HKMMS). *Social Psychology and Psychiatric Epidemiology, 50*(9), 1379-1388. <http://doi.org/10.1007/s00127-015-1014-5>

Lu, S., Liu, T., Wong, G. H. Y., Leung, D. K. Y., Sze, L. C. Y., Kwok, W. W., Knapp, M., Lou, V. W. Q., Tse, S., Ng, S. M., Wong, P. W. C., Tang, J. Y. M., Lum, T. Y. S. (2021). Health and social care service utilisation and associated expenditure among community-dwelling older adults with depressive symptoms. *Epidemiology and Psychiatric Sciences,30*, e10, 1-10. <http://doi.org/10.1017/S2045796020001122>

The Chief Executive’s 2021 Policy Address (2021). *Building a bright future together.*  <https://www.policyaddress.gov.hk/2021/eng/pdf/PA2021.pdf>

Witlox, M., Garnefski, N., Kraaij, V., de Waal, M. W. M., Smit, F.., Bohlmeijer, E., & Spinhoven, P. (2021). Blended acceptance and commitment therapy versus face-to-face cognitive behavioral therapy for older adults with anxiety symptoms in primary care: Pragmatic single-blind cluster randomized trial. *Journal of Medical Internet Research, 23*(3), e24366. <https://doi.org/10.102196/24366>

Supplementary Table 2. Thematic analysis results of the online survey on the current mental healthcare system in Hong Kong

| Current system | Observed situation | Suggestion 1 | Suggestion 2 |
| --- | --- | --- | --- |
| 1. Shortage of manpower in mental health service | “From my knowledge, a comprehensive mental health system has already been established, but the current manpower cannot fully respond to the steadily rising service demands.”  “Current primary health care system for mental health service is extremely insufficient. Older adults can only rely on themselves (Self-help), and they will only seek medical help when the situation becomes too severe and being reminded by their family members.” | **Utilise social support**  “User experience or patients’ self-help, mutual help can be an unlimited resource.”  “Human touch is essential in addition to IT support”  “More resources and service setting in community, rather than hospitalization or long-term medical treatment.” |  |
| 2. Low mental health literacy and a high level of stigma | “Carers might not have sufficient mental health knowledge on understand the older adults’ mental distress and primarily seek help from general practitioners. It is essential to proactively discuss ways to involve general practitioner as one of the stakeholders.”  “Currently, there are more medical referral options to psychiatric departments than before, but many obstacles remain. To me, one big challenge is that older adults are unwilling to see a doctor, or caregivers do not understand the severity of the problem. This phenomenon is because (that they think) having depression or suicidal attempt is something shameful.” | **Mental health Training**  “Mental health care needs further development, including a "train the trainers" program for frontline caregivers, social workers, therapists, and general practitioners.”  “The current older adult care workers lack relevant training and supervision. Cooperation with different stakeholders in the district can be strengthened.”  “Mental health knowledge should be a core competence for all frontline workers and professionals in elderly service.”  “One of the strategies to handle limited mental health professionals and services available is to enhance training and support for caregivers, particularly for those facing early stages of mental illness onset.” | **Stigma Reduction**  “A desirable primary mental health care system should demonstrate stigma free in getting mental health service.”  “An ideal primary mental health care system for older adults can cover different social sectors and even the whole community. Apart from treating individuals with common mental disorders, it can also collaborate with education and social media to provide prevention and advocacy work. This system would help educate society members (possibly caregivers or future elderly) on basic mental health knowledge for the elderly and work towards destigmatizing various mental disorders.” |
| 3. Fragmented service and many hurdles to receiving services | “The impression is that overall, HK does have a variety of services for older people in need, at least on paper. However, the system is clumsy, and there may be a lot of overlapping services used by frequent service users, (who are) just a small portion of people in need.”  “The mental health care system for older people is focused on service mismatch for the elderly.” | **Relevant service**  “Approximately 1 in 9 older people in Hong Kong suffers from a common mental disorder, and treatment and health education can be provided according to the varying degrees of severity.”  “The most convenient approach to handle case matching and referral is to start with the district elderly community centre in the 18 districts. If the centre's social workers can refer them to local family doctors or specialists for intervention based on individuals’ severity and needs. “  “Service matching and efficient referrals should be essential, particularly for older people, in view of the limited mental health professionals and services available.”  “Protocols of intervention and referral mechanisms to be in place at elderly services”  “Specialized intervention approaches at community mental health services to pick up referrals, while applying evidence-based models for effective and efficient intervention.” | **Early intervention**  “Early detection and intervention for elderly mental health require easy access and user-friendliness. Therefore, at existing elderly touchpoints, including neighbourhood elderly centres, district elderly centres, and clinics, training frontline staff or having mental health centre staff provide services through outreach in elderly care is essential.”  “When mental health care is concerned, early identification, service matching and efficient referrals should be essential, particularly for older people, in view of the limited mental health professionals and services available”  “Partnership between elderly and mental health services, with prevention and early identification at elderly services.” |
| 4. Long waiting time to receive service, inadequate involvement of the private sector | “The combination treatment of medication and evidence-based psychosocial intervention is more effective and reduces the relapse rate of mental illness, especially depression. However, it may not be available routinely for patients and their carers, and patients need to wait for a long time to receive services.” | **Sustainable Care**  “Arranging regular visits from psychiatric nurses or doctors to elderly centres or providing home services can be more easily accepted by the elderly than going to clinics or hospitals.”  “Comprehensive support is needed for elderly mental health services, with care managers, case managers, or mental health coaches following up, rather than just providing short-term professional intervention. Follow-up frequency and duration should be determined based on the level of risk.”  “Consider setting up a visiting medical cum social service team for the treatment and maintenance of older people in the community instead of just dealing with acute cases.” | **Shorten Waiting time**  “Suggest to involve more participation from private psychiatrists/clinical psychologists. I am wondering if the private healthcare can provide faster support and the case is assessed as an urgent new condition, could funding methods such as medical vouchers or public-private partnerships be provided to shorten waiting times and reduce the burden on public healthcare?”  “We need clear longer-term planning for serving the older population as soon as possible so that the whole society can prepare for providing the right care services for them.” |
| 5. No long-term policies on elderly mental health care | “The current primary health care system primarily focused on physical health.”  “I agree that the current government policy does not prioritize older adult mental health issues. Although the policy focuses on healthcare and aging in place, physical health and mental health mutually affect each other. Therefore, a comprehensive aging policy should address both aspects simultaneously.”  “Based on my observations, most resources for older adult services are allocated to district elderly community centres. The role of neighbourhood elderly centres in supporting elderly mental health can be enhanced.” | **Integration**  “In primary care for the elderly, it is crucial to explore how to integrate the support services of physical and mental health, with case managers should make appropriate service matches and coordination.”  “Ideally speaking, an accessible and one-stop service that can streamline many services available in the community.”  “Suggest integrating mental health with existing primary care system for elderly.”  “Integrate and combine the services of Hospital Authority's Integrated Mental Health Program (IMHP), district health centre, district elderly community centre, mental health care centre, various NGOs, and self-help organisations to form a grassroots mental health service network for the elderly.”  “Set up a case management system of mental health services across all related organisations, NOT just confined to individual medical or social services” | **Recovery model**  “Recovery-oriented mental health practice … is crucial for engagement to treatment and sustaining recovery. The service delivery may emphasise empowerment/ enablement of individuals and their carers in their illness management proactively as treatment partners for better outcomes such as remission, staying well and/or wellbeing.” |
